# A Comparative Genomic Assessment of Invasive Potential in the Planthopper *Orosanga japonica* (Hemiptera: Ricaniidae)

**DOI:** 10.3390/insects17090932

**Published:** 2026-09-05

**Authors:** Yusuf Ulaş Çınar, Mehmet Ali Balcı, Onur Obut, Tuana Öğretici, Selahattin Barış Çay, Hüsna Yağmur Dural, Fatih Dikmen, Yakup Bakır, Vahap Eldem

**Affiliations:** 1 Institute of Graduate Studies in Sciences, Istanbul University, Istanbul 34134, Türkiye; yusufulascinar@ogr.iu.edu.tr (Y.U.Ç.); balci.mehmetali@ogr.iu.edu.tr (M.A.B.); onur.obut@ogr.iu.edu.tr (O.O.); tuanaogretici@ogr.iu.edu.tr (T.Ö.); selahattinbariscay@ogr.iu.edu.tr (S.B.Ç.); husnayagmurdural@ogr.iu.edu.tr (H.Y.D.); 2 Department of Biology, Faculty of Science, Istanbul University, Istanbul 34134, Türkiye; fatih.dikmen@istanbul.edu.tr; 3 Department of Medical Laboratory Techniques, Vocational School of Health Services, Atlas University, Istanbul 34403, Türkiye

**Keywords:** *Orosanga japonica*, invasive species, gene family evolution, positive selection, genome annotation, detoxification

## Abstract

*Orosanga japonica* (Hemiptera: Ricaniidae) is an invasive, polyphagous planthopper established in Türkiye since 2007 that threatens economically important perennial and field crops across the Black Sea region. Although its establishment and rapid spread suggest considerable adaptive capacity, the genomic basis of this potential remains unexplored. We combined whole-genome sequencing with RNA-Seq data to generate a functionally annotated genome, recovering 92.5% of conserved single-copy orthologs. Utilizing these resources, we performed comparative genomic analyses across 20 additional hemipteran and thysanopteran species, investigating gene family dynamics and genome-wide positive selection. These analyses revealed 52 gene families with statistically significant, lineage-specific size changes (24 expansions and 28 contractions): expanded families were enriched for C2H2 zinc-finger transcription factors, α/β-hydrolases, major facilitator transporters, serpins, and chemosensory proteins, whereas contracted families included a cytochrome P450 family. Additionally, branch site tests identified 122 candidate genes under lineage-specific positive selection. These loci fall into three functional categories potentially associated with invasion-related traits: host detection and neuronal excitability, stress tolerance (xenobiotic, oxidative, and proteostatic), and barrier remodeling. Together, these genomic signatures highlight candidate molecular mechanisms that may contribute to host detection, xenobiotic tolerance, and ecological plasticity, providing a framework for evolutionary analysis and the targeted management of this emerging agricultural pest.

## Simple Summary

The planthopper *O. japonica* is an insect native to Asia that has invaded Türkiye, where it spreads through the Black Sea region and damages valuable crops such as tea, hazelnut, and kiwifruit by feeding on plant sap; it is now beginning to invade Southern Europe as well. Because it thrives in new environments and chemical control is often limited, scientists need to understand what makes it such a successful invader. In this study, we read the insect’s genetic material and examined which of its genes are active, then compared them with those of related insects. We identified candidate genes potentially linked to finding and feeding on host plants, tolerating plant toxins and environmental stress, and strengthening the insect’s protective outer barrier. These genes may help explain its invasive success and could serve as targets for more precise, environmentally friendly control methods to help protect crops.

## 1. Introduction

*Orosanga japonica* (Melichar, 1898) (Hemiptera: Ricaniidae), a polyphagous planthopper of the superfamily Fulgoroidea, is native to Eastern and Central Asia but has progressively expanded across the Palearctic, where it is now recorded from China, Korea, and Japan through to Bulgaria, Georgia, and Azerbaijan [1,2,3,4]. Like other Ricaniidae, it is a damaging pest of both forest and agricultural systems: it exploits an unusually broad range of host plants, reproduces prolifically, coats foliage in honeydew, deposits conspicuous egg masses in woody tissue, and can act as a vector of plant disease [5,6,7]. In Türkiye, the species was first detected at Rize in 2007 and within a few years had become established throughout the humid, densely vegetated Eastern Black Sea region; its arrival is generally attributed to the movement of nursery stock from neighboring Georgia [8,9]. In early Turkish publications, the species was repeatedly misidentified as *Ricania simulans* [10,11,12], reflecting a complex nomenclatural history: originally described as *Ricania japonica* [13] and long classified within *Ricania* [3,13,14], it was later reassigned to the genus *Orosanga* following successive taxonomic revisions [15,16,17,18]. Both nymphs and adults feed indiscriminately on vegetables, shrubs, and trees, draining sap from stems, leaves, and fruit [12]. Although quantitative estimates of yield loss and economic impact are not yet available for the invaded range, the species is regarded as a threat to the region’s principal perennial crops, particularly tea, hazelnut, and kiwifruit, as well as to a wide range of field and orchard plants such as beans, aubergine, cabbage, blackberry, fig, grape, maize, and pepper [12,19].

Chemical control of *O. japonica* is constrained by the same considerations that increasingly limit insecticide use more broadly, namely the risks to human health and non-target organisms together with the evolution of resistance [20]. Within the region’s certified-organic tea plantations, synthetic pesticides cannot be applied at all [21]. These limitations have heightened interest in more sustainable alternatives, including biotechnical and semiochemical approaches [22,23], biological control [24,25,26], and the design of more selective insecticides [27,28]. Each of these strategies ultimately depends on molecular knowledge of the pest: semiochemical approaches require characterization of its chemosensory gene repertoire, selective insecticides and RNAi-based methods require well-annotated candidate target genes, and resistance management requires an inventory of its detoxification-related gene families. Genomic resources of the kind generated in this study are therefore a shared prerequisite for these avenues.

Genome sequencing has transformed the study of population expansion, invasion, and adaptation in agricultural insects [29,30,31]. Comparative and functional genomics have identified new control targets and informed integrated pest management, clarified insecticide target site mutations [32], enabled the design of more selective chemistries [33], illuminated the detoxification pathways that underlie resistance [34], linked host plant adaptation to resistance [35,36], and revealed olfactory targets for behavioral manipulation [37,38,39]. A well-annotated genome is, moreover, a prerequisite for precise gene editing approaches such as CRISPR-Cas9 [40,41]. Comparative genomic studies of invasive insects frequently report gene family expansions concentrated in functions that mediate interaction with the external environment, such as detoxification, chemosensation, and stress response, which may provide raw material upon which selection can act [42]; this pattern is not universal, however, and gene family expansion should not be regarded as a general prerequisite for invasion success. Positive selection can, in turn, fine-tune individual loci: across taxonomically diverse invaders, positively selected genes are repeatedly enriched for functions related to stress response [43], detoxification [44], oxidation reduction [45], and reproduction [46]. Two caveats frame the interpretation of such comparative analyses. First, signatures of gene family change and positive selection detected along a focal species lineage accumulate over long evolutionary timescales; they characterize the evolutionary background of the species and cannot, by themselves, be attributed to a recent invasion event. Second, demonstrating that any candidate gene contributes to invasion success ultimately requires functional validation or comparisons between invasive and native populations. For *O. japonica*, even the baseline molecular information needed to formulate such hypotheses has been lacking: no functional genome annotation existed for the species, and neither its gene family dynamics nor its selection landscape had been characterized.

In the present study, we characterize the genomic background of *O. japonica* through an integrated comparative genomic approach. We generated short- and long-read whole-genome sequencing data from a specimen collected in the invasive Turkish range, providing the first genomic data for this population, together with stage-resolved RNA-Seq data from nymphs and adults. A chromosome-level reference assembly for the species (ihOroJapo4.1) had recently become available through long-read sequencing; because this assembly lacked functional annotation, which limited its value for evolutionary inference, we used it as the genomic baseline for our analyses. Our specific objectives were to: (i) generate an RNA-Seq-supported functional annotation of the *O. japonica* genome, the first available for the species; (ii) characterize statistically significant, lineage-specific gene family expansions and contractions; and (iii) identify candidate genes under lineage-specific positive selection. Together, these analyses provide the first genome-wide annotation and comparative genomic characterization for any member of the family Ricaniidae and yield a set of candidate genes and gene families whose possible contributions to invasion-related traits can be tested in future functional and population genomic studies.

## 2. Materials and Methods

### 2.1. Sampling

Nymph and adult individuals of *O. japonica* were collected from the Atatürk Urban Forest in Istanbul (41°8′4.49′′ N, 29°1′56.34′′ E) using traps. Field collection was conducted under a research permit issued by the Ministry of Agriculture and Forestry, General Directorate of Nature Conservation and National Parks (Permit Number: E-21264211-288.04-7094785). Collected specimens were placed directly into 50 mL Falcon tubes and transported to the Istanbul University Molecular Biodiversity Laboratory. Upon arrival, both adult and nymph samples were immediately stored at −80 °C to preserve tissue integrity for subsequent DNA and RNA extraction.

### 2.2. HMW DNA Extraction and Whole-Genome Sequencing

High-molecular-weight (HMW) genomic DNA was extracted from a single adult *O. japonica* specimen using the NucleoBond HMW DNA kit (Macherey-Nagel, Düren, Germany), following the manufacturer’s protocol. In brief, the specimen was lysed in 5 mL of H1 buffer and homogenized with an IKA-RW20 digital mixer. Proteinase K (200 µL) was then added and the lysate incubated at 50 °C for 30 min, after which 100 µL of RNase A was added and incubated for a further 5 min at room temperature. The DNA was bound to a NucleoBond HMW column pre-equilibrated with 12 mL of H2 buffer, washed with 6 mL of H3 buffer and, after removal of the filter, washed with 12 mL of H4 buffer and eluted in 5 mL of H5 buffer. The eluate was precipitated with isopropanol, washed with 70% ethanol, and resuspended in 150 µL of HE buffer. DNA concentration was determined with a Qubit 4 fluorometer (Thermo Fisher Scientific, Waltham, MA, USA), and integrity was assessed by gel electrophoresis. Short-read libraries were prepared using the VAHTS Universal DNA Library Prep Kit for MGI (Vazyme, Nanjing, China) and sequenced on the DNBSEQ-G400 platform (MGI Tech, China), generating 150 bp paired-end reads. For long-read sequencing, a library was constructed with the SQK-LSK114 ligation sequencing kit and sequenced on an R10.4 flow cell using a MinION Mk1C instrument (Oxford Nanopore Technologies, Oxford, UK). The genomic reads generated here were used for molecular species identification (Section 2.3) and are provided as an independent genomic resource for the invasive Turkish population (BioProject PRJNA1504096); as detailed in Section 3.1, all downstream comparative analyses were performed on the publicly available chromosome-level assembly ihOroJapo4.1 rather than on a newly generated assembly. Read counts, sequencing yields, and base quality statistics for both platforms are reported in Tables S1 and S2.

### 2.3. Molecular Species Identification

To confirm the identity of the specimen, mitochondrial scaffolds were assembled from the short-read data using GetOrganelle v1.7.7.0 [47] and annotated with GeSeq v2.03 on the Chlorobox web server [48]. The complete mitochondrial genome was then queried against the NCBI nucleotide database using BLASTn v2.17.0, and the cytochrome c oxidase subunit I (COI) barcode region extracted from the mitogenome was independently searched against the Barcode of Life Data system (BOLD) to provide an additional line of confirmation for the species assignment.

### 2.4. Transcriptome Library Preparation and Sequencing

Total RNA was extracted separately from three adults and three nymphs, each specimen serving as an independent biological replicate, using TRIzol reagent (Invitrogen, Carlsbad, CA, USA) following homogenization on a MagNA Lyser (Roche, Mannheim, Germany). Phases were separated with chloroform, and RNA was precipitated with isopropanol, washed in 75% ethanol, and dissolved in 30 µL of RNase-free water. RNA concentration and purity were assessed on a NanoDrop ND-2000c spectrophotometer (Thermo Scientific, Wilmington, DE, USA), and RNA integrity was evaluated on a QIAxcel capillary electrophoresis system (Qiagen, Hilden, Germany); only samples with an OD_260_/_280_ ratio of approximately 2.0 and an RNA integrity score (RIS) above 7.0 were retained for library construction. For each biological replicate, a strand-specific (directional) cDNA library was prepared using the MGIEasy RNA Directional Library Prep Kit (MGI Tech, Shenzhen, China). Messenger RNA was first enriched from total RNA, fragmented, and reverse-transcribed into double-stranded cDNA, with strand orientation preserved through the incorporation of dUTP during second-strand synthesis. The cDNA was then end-repaired, 3′-adenylated, and ligated to sequencing adapters; the resulting fragments were circularized and subjected to rolling-circle amplification to generate DNA nanoballs (DNBs). Library quality and concentration were verified using a QIAxcel^®^ DNA High Resolution Kit, and paired-end sequencing (2 × 150 bp) was performed on the DNBSEQ-G400 platform (MGI Tech, Shenzhen, China). Raw reads were adapter- and quality-trimmed with Trimmoatic v.040 [49], and per-sample statistics of the resulting clean reads are reported in Table S5.

### 2.5. Genome Annotation

Because a high-quality, chromosome-level long-read assembly (ihOroJapo4.1) was already publicly available but lacked functional annotation, our contribution focused on annotating this genome. Structural and functional gene prediction were carried out with NCBI EGAPx v0.5.2 (https://github.com/ncbi/egapx, accessed on 19 May 2026), the public release of the NCBI Eukaryotic Genome Annotation Pipeline, using the assembly together with the RNA-Seq evidence described above. Hemiptera (NCBI Taxonomy ID 7524) was provided as the taxonomic context, allowing EGAPx to select appropriate Hemiptera- and Insecta-specific protein evidence sets and hidden Markov models (HMMs) [50]. The completeness and consistency of the resulting annotation were then assessed with OMArk v0.5.0 [51] against the Paraneoptera conserved ortholog set.

### 2.6. Gene Family Expansion and Contraction Analysis

The proteomes of 20 well-annotated comparator species were retrieved from NCBI datasets: *Frankliniella occidentalis* (GCA_000697945.5), *Thrips palmi* (GCA_012932325.1), *Bemisia tabaci* (GCA_918797505.1), *Cyamophila willieti* (GCA_041222665.1), *Planococcus citri* (GCA_950023065.1), *Kerria lacca* (GCA_045014175.1), *Aphis gossypii* (GCA_020184175.2), *Rhopalosiphum maidis* (GCA_003676215.3), *Rhopalosiphum padi* (GCA_020882245.1), *Semiaphis heraclei* (GCA_046119115.1), *Acyrthosiphon pisum* (GCA_005508785.2), *Metopolophium dirhodum* (GCA_019925205.1), *Ranatra chinensis* (GCA_040954505.1), *Nezara viridula* (GCA_928085145.1), *Nesidiocoris tenuis* (GCA_036186465.1), *Apolygus lucorum* (GCA_009739505.2), *Rhynocoris fuscipes* (GCA_040020575.1), *Nilaparvata lugens* (GCA_014356525.1), *Lycorma delicatula* (GCA_047948215.1), and *Homalodisca vitripennis* (GCA_021130785.2). These species were selected to provide broad taxonomic coverage of the Hemiptera (Sternorrhyncha, Heteroptera, and Auchenorrhyncha, including the fulgoroid relatives *N. lugens* and *L. delicatula*), with two thysanopteran species serving as outgroups, while restricting the panel to uniformly high-quality, NCBI-annotated proteomes so that inferred gene family size differences reflect biology rather than heterogeneous annotation quality. To standardize the datasets, the longest protein isoform of each gene was retained with AGAT v1.4.2 (agat_sp_keep_longest_isoform.pl) [52], and orthogroups and single-copy orthologs were inferred with OrthoFinder2 v2.5.5 under default settings [53]. Alignments of the single-copy orthologs (OrthoFinder -m MSA) were concatenated for phylogenetic reconstruction [54], and the species tree was inferred with IQ-TREE v3.0 under the Q.insect + R5 model with 1000 bootstrap replicates [55]. Following Yang et al. [56] and Ouyang et al. [57], the resulting tree topology and branch lengths were used to model gene family size change under a random birth-and-death process in CAFE5 v1.1.0 [58]. To account for potential inaccuracies in gene copy number estimation arising from genome assembly and annotation errors, an error model was incorporated into the CAFE5 analysis (-e option; maximum family size = 170, with an estimated error probability of 0.0153 for a deviation of ±1 gene copy). Families with *p* < 0.05 were considered to have undergone significant size change, and families showing significant change along the terminal branch leading to *O. japonica* were classified as lineage-specific expansions or contractions according to the sign of the copy number change inferred on that branch relative to the reconstructed ancestral state.

### 2.7. Genome-Wide Positive Selection Analysis

Genome-wide positive selection was tested following the framework of Álvarez-Carretero et al. [59]. Of the single-copy orthogroups identified above, 2318 were retained for analysis. Proteins and their corresponding coding sequences were aligned at the codon level with PAL2NAL v14.1 [60], and a maximum-likelihood gene tree was inferred for each orthogroup with IQ-TREE v3.0 [55]; per-orthogroup gene trees were used in the codon analyses to accommodate potential gene-tree/species-tree discordance. To remove ambiguously aligned regions while preserving the codon reading frame, multiple sequence alignments were processed using trimAl v1.5.1. [61] with the -automated1 heuristic and -backtrans parameters. The codon alignments and their gene trees were then analyzed with the codeml program in PAML v4.10.7 [62] using branch site models, with *O. japonica* designated as the foreground branch. For each orthogroup, branch site model A, which allows a class of sites with ω > 1 on the foreground branch, was compared against the corresponding null model in which this site class is constrained to ω = 1, and statistical significance was assessed by likelihood-ratio test with one degree of freedom. The resulting *p*-values were corrected for multiple testing across all 2318 tested orthogroups using the Benjamini–Hochberg false discovery rate (FDR), with an adjusted threshold of padj < 0.05; genes significant at the branch level were further screened for individual positively selected sites using Bayes Empirical Bayes (BEB) posterior probabilities, with sites at BEB > 0.95 beingregarded as high-confidence. Gene symbols and product names follow the corresponding NCBI RefSeq orthologs; the LOC identifiers reported in the text are therefore RefSeq orthogroup representatives (predominantly from *Acyrthosiphon pisum*) used for annotation transfer and do not denote *O. japonica* gene models, which are listed alongside them in Table S7. Gene Ontology (GO) over-representation analysis of the positively selected genes was performed across the biological process, molecular function, and cellular component domains (FDR < 0.05), using all single-copy orthologs as the background gene set, and heatmaps and enrichment plots were generated in R using pheatmap v1.0.12 [63] and ggplot2 v3.5.1 [64].

### 2.8. Differential Expression and Transcription Factor Analysis

To characterize stage-specific gene expression, the clean RNA-Seq reads from the adult and nymph samples (three biological replicates each) were aligned to the ihOroJapo4.1 reference genome with HISAT2 v2.2.3 [65], using the EGAPx annotation to guide spliced alignment; overall alignment rates per sample are reported in Table S3. Aligned reads were assembled and quantified with StringTie v3.0.3 [66], and gene-level read counts were generated with featureCounts (Subread package v2.1.1) [67] on the basis of the reference annotation. Differential expression between the adult and nymph stages was assessed with DESeq2 v1.50.2 [68], and genes with an absolute log2 fold change ≥1 and a Benjamini–Hochberg-adjusted *p*-value < 0.05 were considered differentially expressed genes (DEGs). For visualization, normalized expression values were transformed to per-gene Z-scores and displayed as heatmaps using the EnrichedHeatmap package in R [69]. Transcription factors were identified by searching the predicted proteome against transcription factor family profiles using hidden Markov models (HMMs). The DNA-binding-domain HMM profiles compiled in the Animal Transcription Factor Database (AnimalTFDB4) [70] resource were searched against the *O. japonica* proteins with hmmsearch v3.4 [71], and candidate transcription factors were assigned to families according to the database’s family classification rules. Transcription factors among the stage-specific DEGs were then classified by family and visualized as adult-biased and nymph-biased expression heatmaps.

## 3. Results

### 3.1. Genome Sequencing, Species Identification, and Reference-Assembly Selection

Whole-genome sequencing of a single Turkish *O. japonica* specimen produced complementary long- and short-read datasets. Long-read sequencing with the SQK-LSK114 kit on a single R10.4 MinION flow cell generated 727,605 reads totaling 6.59 Gb of raw sequence, with a mean read length of 9063 bp (median 2534 bp), a read-length N50 of 31,857 bp, and a mean read quality of Q13.3 (median Q14.1), consistent with the accuracy expected from R10.4 chemistry (Table S1). Short-read sequencing on the DNBSEQ-G400 platform yielded 318,047,568 paired-end reads (95.41 Gb of 150 bp paired-end data); base quality was high, with 97.41% and 95.67% of bases reaching Q20, and 90.70% and 87.55% reaching Q30 in the forward and reverse reads, respectively, and GC content ranged from 32.84% to 33.04% (Table S2). The long- and short-read data generated here were initially aimed at improving and extending the genomic resources available for *O. japonica*. During the course of this study, however, a chromosome-level reference assembly for the species (ihOroJapo4.1) was independently released. Given the high contiguity and completeness of this reference, and the comparatively modest long-read coverage obtained here, we chose to proceed with the existing assembly as the genomic baseline for all downstream analyses rather than generate a lower-quality, redundant assembly. Importantly, because the specimen sequenced here was collected from the invasive Turkish population, these data nonetheless represent a valuable and independent genomic resource for this range to support future population-level and comparative studies.

To confirm the taxonomic identity of the sequenced specimen before proceeding with downstream analyses, and given the history of misidentification within this group, species assignment was verified using a barcode-based reference framework. The cytochrome c oxidase subunit I (COI) region, extracted from the mitochondrial genome assembled from the short-read data, was queried against the Barcode of Life Data system (BOLD) using the Species Identification engine. The search returned *O. japonica* as the top match at 99.85% similarity, well above the standard BOLD threshold for confident species-level assignment (>98% identity) (Table S4).

### 3.2. Transcriptome Sequencing, Genome Annotation, and Proteome Assessment

Transcriptome sequencing of adult and nymph stages, each in three biological replicates and sequenced as paired-end reads, produced 159,914,636 clean reads after quality control, totaling approximately 21.9 Gb of 150 bp sequence. Per-sample base quality was high, with Q20 and Q30 values ranging from 95.4% to 97.1% and from 88.7% to 91.9%, respectively (Table S5). These reads were used as transcriptional evidence for genome annotation. EGAPx annotation of the ihOroJapo4.1 assembly (1.50 Gb across 12 chromosomes and 184 unplaced scaffolds; 31.93% GC), guided by the RNA-Seq evidence and Hemiptera-specific models, yielded 15,243 gene models, of which 12,402 were protein-coding and the remainder non-coding, including 1718 transfer RNA, 784 long non-coding RNA, 236 ribosomal RNA, and smaller numbers of small nuclear and small nucleolar RNA genes (Table 1). The protein-coding genes gave rise to 18,008 mRNA transcripts and 17,652 predicted proteins, with an average protein length of 546.5 amino acids and an average exon length of 244.35 bp across 166,644 exons. This constitutes, to the best of our knowledge, the first functional annotation of the *O. japonica* genome, directly complementing the previously unannotated long-read assembly.

**Figure 1:**
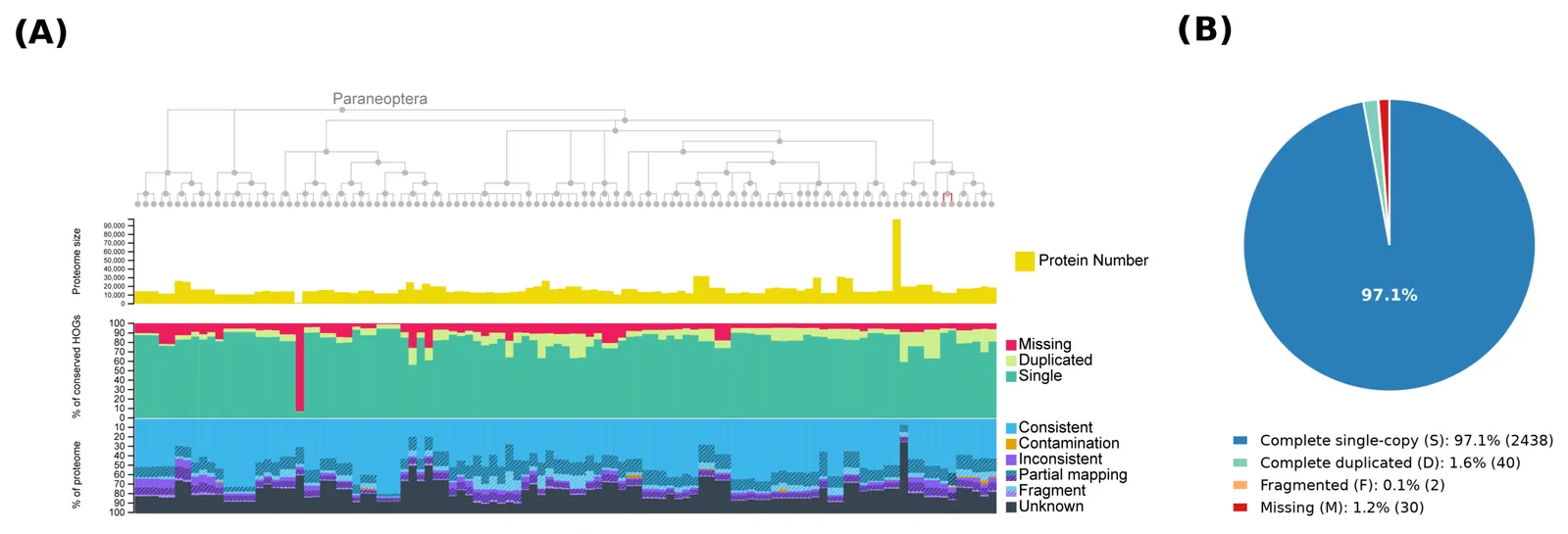
Comparative proteome assessment and gene space completeness of the annotated genome. (**A**) Phylogenetic placement of the focal species within Paraneoptera. From top to bottom, the tracks show: the reference species tree; proteome size (predicted protein number per species, gold bars); the distribution of conserved HOGs classified as single-copy (single), duplicated, or missing; and the proportion of each proteome assigned to quality categories (consistent, contamination, inconsistent, partial mapping, fragment, unknown). The focal species is highlighted in red. (**B**) BUSCO completeness of the predicted proteome assessed against the hemiptera_odb10 lineage dataset (*n* = 2510 orthologs; proteins mode, BUSCO v5.7.1).

The completeness and reliability of the annotation were evaluated using two complementary benchmarks (Figure 1). A proteome-based assessment with OMArk against the Paraneoptera reference lineage indicated high completeness. Of the 4585 conserved hierarchical orthologous groups (HOGs) expected for this clade, 92.45% (4239) were recovered as single-copy and 2.14% (98) as duplicated, while only 5.41% (248) were missing, corresponding to an overall completeness of 94.59% (Figure 1A). At the proteome level, 71.49% of the 12,402 assessed proteins were consistent with the expected lineage placement, 12.93% inconsistent, and 14.36% of unknown placement, with potential contaminants accounting for only 1.23% (152 proteins). Species composition analysis assigned the majority of query proteins to Cimicomorpha (61.47%, 7624), consistent with the hemipteran origin of the assembly, and detected a single minor fungal contaminant signal (Pezizomycotina; 1.30%, 161 proteins), which was excluded from downstream interpretation (Table S6). A complementary, BUSCO-based assessment against the Hemiptera lineage was fully concordant, recovering 98.7% of conserved orthologs as complete (97.1% single-copy (2438) and 1.6% duplicated [40]), with only 0.1% fragmented (2) and 1.2% missing (30), indicating a near-complete gene set (Figure 1B). Together, these concordant OMArk and BUSCO results confirm a highly complete, high-quality annotation and provide a reliable foundation for the comparative gene family and positive selection analyses that follow.

### 3.3. Dynamic Gene Family Evolution in the O. Japonica Lineage

**Figure 2:**
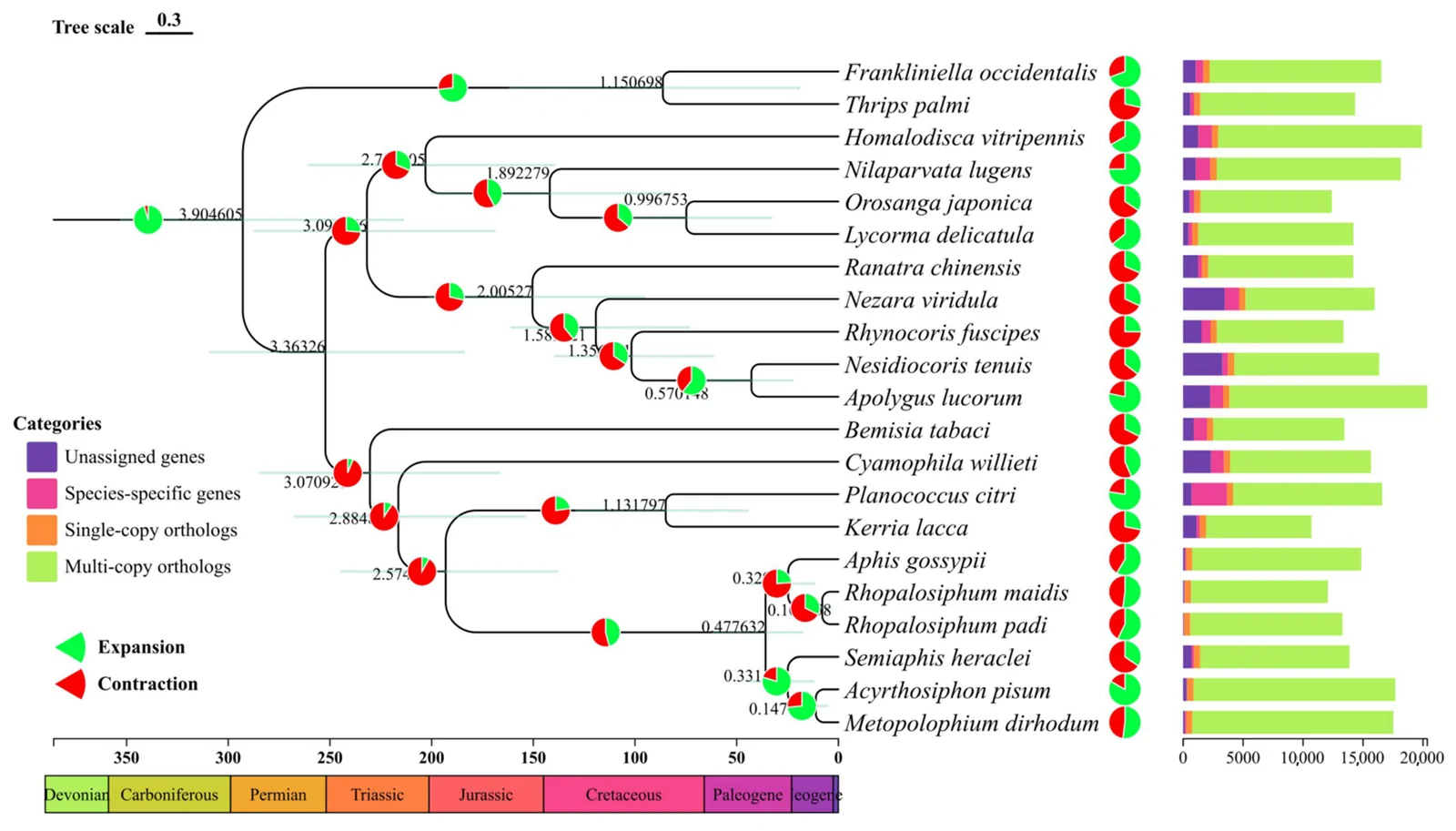
Phylogenetic relationships and gene family evolution across 24 hemipteran and outgroup species. The time-calibrated species tree (divergence times in Mya, geological timescale below) was inferred from single-copy orthologs. Pie charts at each node and tip indicate the proportion of expanded (green) and contracted (red) gene families estimated by CAFÉ. Stacked bars (right) show the gene composition of each species: unassigned genes, species-specific genes, single-copy orthologs, and multi-copy orthologs. Tree scale = 0.3 substitutions per site.

Orthology inference across the analyzed species yielded a total of 40,804 orthogroups, which were used both to reconstruct a time-calibrated species phylogeny from single-copy orthologs and to model the dynamics of gene family evolution (Figure 2). In the resulting phylogeny, *O. japonica* was recovered within the Fulgoroidea as the closest relative of the spotted lanternfly *Lycorma delicatula*, together forming a clade with the planthopper *Nilaparvata lugens* and, more broadly, with the sharpshooter *Homalodisca vitripennis*, in agreement with current Auchenorrhyncha systematics. On the time-calibrated tree, *O. japonica* and *L. delicatula* were estimated to have diverged approximately 100 million years ago, during the mid-Cretaceous, within a Fulgoroidea lineage that had separated from *N. lugens* by around 190 million years ago (Figure 2). Mapping of gene category composition onto the tree showed that, as in the other hemipterans analyzed, the majority of *O. japonica* genes were assigned to multi-copy orthologous groups, with smaller proportions of single-copy orthologs and of species-specific and unassigned genes (Figure 2). Superimposing gene family size changes onto this framework, CAFE analysis identified 474 families whose size changed significantly along the species tree (*p* < 0.05), represented as expansion (green) and contraction (red) events at each node in Figure 2. Of these, 52 were significant specifically in the *O. japonica* lineage, comprising 24 expanded and 28 contracted families (Figure 2; Table S7). Because these size changes are inferred along the entire terminal branch of the species, they reflect gene family evolution accumulated over the long-term history of the *O. japonica* lineage rather than changes specifically associated with its recent invasion of Türkiye.

**Figure 3:**
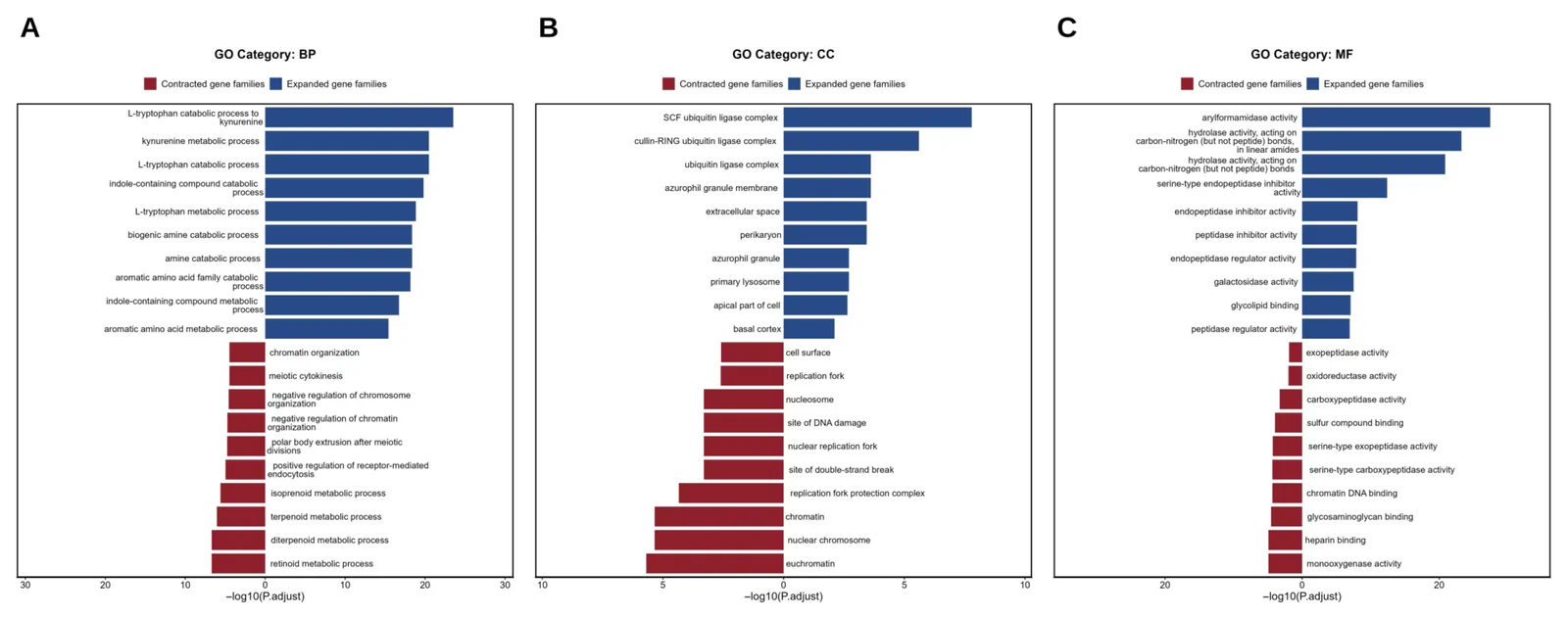
The GO enrichment analysis of expanded and contracted gene families. The bidirectional bar plots display the top 10 significantly enriched GO terms (adjusted *p* < 0.05) in each direction across three categories: (**A**) biological process (BP), (**B**) cellular component (CC), and (**C**) molecular function (MF). The x-axis represents the statistical significance of the enrichment, denoted as −log_10_ (p.adjust). Blue bars directed to the right indicate pathways enriched in expanded gene families, whereas dark red bars directed to the left indicate pathways enriched in contracted gene families.

GO enrichment analysis distinguished the functional signatures of the expanded and contracted families (Figure 3). Expanded families were significantly enriched (*q* < 0.05) for biological processes centered on tryptophan and kynurenine catabolism and on aromatic amino acid and biogenic amine catabolism (Figure 3A); for molecular functions including arylformamidase activity, hydrolase activity acting on carbon–nitrogen bonds in linear amides, peptidase- and endopeptidase inhibitor activity, and galactosidase activity (Figure 3C); and for cellular components corresponding to SCF and cullin-RING ubiquitin ligase complexes (Figure 3B). In contrast, contracted families were enriched for chromatin organization and DNA replication and repair; for isoprenoid, terpenoid, and retinoid metabolism; and for molecular functions including exopeptidase and carboxypeptidase activity, oxidoreductase activity, and monooxygenase activity (Figure 3). This division mirrors the family-level changes described below, in which expansions fall largely on transport, hydrolytic, and protein-regulatory functions while several classical detoxification- and chromatin-associated families are reduced.

The most strongly expanded orthogroups were dominated by transcriptional regulators and mobile element-derived families. The single largest expansion (OG0000326) was composed of C2H2-type zinc-finger domains (PF00096; IPR013087), as were two further large expansions (OG0000754 and OG0006276), together pointing to substantial diversification of C2H2 zinc-finger transcription factors. OG0000208 comprised hAT-family and BED-type zinc-finger domains alongside Hermes transposase and THAP domains, while OG0000069 was characterized by Myb/SANT-like (MADF) and Rad60/SUMO-like domains. The prominence of these regulatory and transposon-associated families indicates that lineage-specific expansion in *O. japonica* has been accompanied by considerable turnover of transcription factor and mobile element content.

Beyond these regulatory families, several expansions corresponded to gene families whose annotated functions represent candidate associations with invasion-related traits, many of which were directly reflected in the enriched GO terms. Multiple orthogroups encoded major facilitator superfamily and sugar-transporter proteins (OG0000335, OG0000142, OG0000110), indicating an enlarged transmembrane transport repertoire. Detoxification- and digestion-related families were likewise expanded, including α/β-hydrolase-fold enzymes bearing ferulic acid esterase domains (OG0000926), glycoside hydrolase family 1 β-glucosidases (OG0000134, consistent with the enriched galactosidase activity), and carbon–nitrogen hydrolase/vanin-like enzymes (OG0000620, consistent with the enriched arylformamidase and carbon–nitrogen hydrolase activities). A chemosensory protein family (insect pheromone-binding/OS-D; OG0000252) was also expanded. Expansion of the serpin family of serine protease inhibitors (OG0000226) accords with the strong enrichment of peptidase and endopeptidase inhibitor activity, while expansion of BTB/POZ-MATH domain proteins (OG0000290), which frequently act as cullin-RING ligase adaptors, is consistent with the enrichment of ubiquitin ligase complexes. Finally, a Methuselah-like G-protein-coupled receptor family (OG0000375), members of which have previously been linked to stress resistance and lifespan regulation in other insects, was also among the expanded families. We emphasize that gene family size changes alone do not demonstrate these functions in *O. japonica*; the possible relevance of these candidate associations to host use, digestion, chemosensation, and stress physiology remains to be established through functional validation.

In contrast, the contracted families were enriched in mobile element-derived and reverse transcriptase-containing orthogroups (OG0000232, OG0000094, OG0000025), in cytochrome P450 (OG0000021, OG0000174), small heat-shock protein/α-crystallin (OG0000353), and in core and linker histone families (OG0000034, OG0000063); the latter is mirrored by the enrichment of chromatin organization and DNA replication terms among the contracted families (Figure 3). Consistent with the cytochrome P450 contraction, monooxygenase and oxidoreductase activities were significantly enriched among the reduced families (Figure 3C). The reduction of cytochrome P450 and small heat-shock protein families, gene classes commonly associated with xenobiotic detoxification and thermal tolerance, indicates that the expansion of metabolic capacity in *O. japonica* is not uniform across all detoxification-related pathways. Rather than a wholesale amplification of detoxification genes, this pattern may instead reflect a streamlining of redundant copies alongside the targeted amplification of specific enzyme families, such as the α/β-hydrolases, transporters, and β-glucosidases noted above.

### 3.4. Branch Site Analyses Identify Candidate Genes Under Lineage-Specific Positive Selection

**Figure 4:**
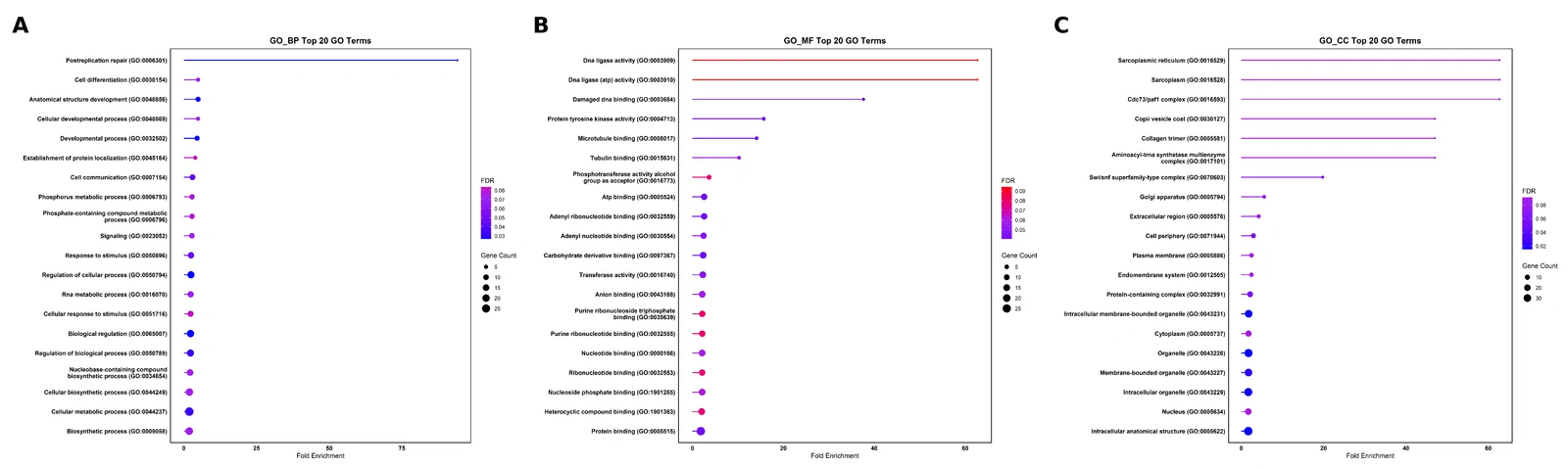
The GO enrichment of positively selected genes in *O. japonica*. Top 20 enriched terms are shown for (**A**) biological process, (**B**) molecular function, and (**C**) cellular component. Lollipop length represents fold enrichment, dot size the number of associated genes, and color the FDR-adjusted *p*-value.

Of the orthogroups inferred across the analyzed species, 2318 single-copy orthogroups were retained and tested for lineage-specific positive selection along the *O. japonica* branch using branch site models in codeml. This analysis identified 122 candidate genes with significant signatures of lineage-specific positive selection (FDR-adjusted *p* < 0.05), of which 95 carried at least one high-confidence positively selected site (Bayes Empirical Bayes posterior probability > 0.95; Table S8). The most significant candidates included the planar cell polarity protocadherin starry night/flamingo (LOC100160214), the neuronal microtubule-associated protein futsch (LOC100158826), zinc-finger protein 608, mucin-3A and mucin-17, and the epidermal transcription factor grainyhead. Functional annotation indicated that the positively selected genes were concentrated in neuronal, developmental, regulatory, and signaling functions rather than in classical detoxification enzymes (Figure 4). GO enrichment recovered biological process terms related to development, cell differentiation, cell communication, and signaling, alongside post-replication and DNA repair processes; the enriched molecular functions were dominated by DNA ligase and damaged DNA-binding activities, protein tyrosine kinase activity, and microtubule and nucleotide binding, while enriched cellular components included the sarcoplasmic reticulum, the SWI/SNF and Cdc73/Paf1 complexes, and the COPII vesicle coat (Figure 4A–C).

**Figure 5:**
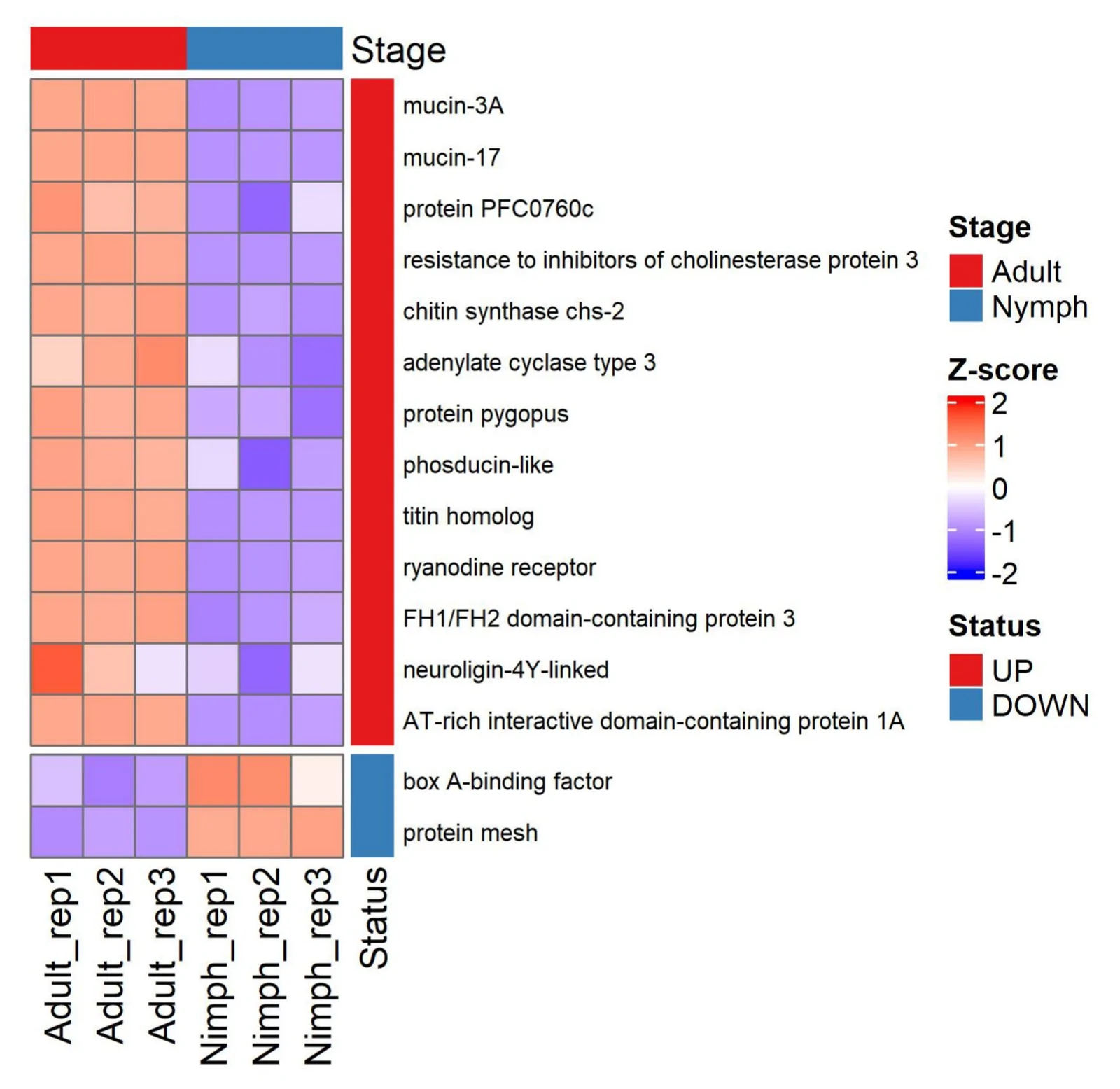
Genes both differentially expressed between developmental stages and under positive selection in *O. japonica*. Rows, genes (protein product names); columns, biological replicates (*n* = 3 per stage). Colors show row-scaled z-scores of normalized expression (red, high; blue, low). Top bar, developmental stage (red, adult; blue, nymph); right bar, direction of change in adults relative to nymphs.

To assess whether these positively selected genes are also transcriptionally dynamic across development, we intersected them with genes differentially expressed between the adult and nymph stages (Figure 5). This intersection was performed as a descriptive screen to flag candidates for follow-up rather than as a test of adaptive function: positive selection acts on protein-coding sequence and may alter protein localization, binding affinity, stability, or turnover without any accompanying change in the timing or level of mRNA expression, so no general correspondence between the two gene sets is expected. Consistent with this, only 15 of the 122 positively selected genes were stage-biased in expression, a modest overlap in the context of the profound, genome-wide transcriptional remodeling that accompanies the nymph-to-adult transition. Thirteen were more highly expressed in adults, including the secreted mucins mucin-3A and mucin-17, the nicotinic-receptor chaperone resistance-to-inhibitors-of-cholinesterase protein 3 (*Ric-3*), chitin synthase *chs-2*, adenylate cyclase type 3, the ryanodine receptor, neuroligin-4, the protein pygopus, and the AT-rich interactive domain-containing protein 1A, whereas two genes, box A-binding factor and the peritrophic-matrix protein mesh, were more highly expressed in nymphs. Although this set is too small for meaningful formal enrichment analysis, the annotations of the named candidates fall qualitatively into three recurrent groups: extracellular matrix and epithelial barrier components (*mucin-3A*, *mucin-17*, *chs-2*, *mesh*), membrane-associated neuronal and signaling proteins (*Ric-3*, the ryanodine receptor, neuroligin-4, adenylate cyclase type 3), and transcriptional or chromatin regulators (pygopus, AT-rich interactive domain-containing protein 1A, box A-binding factor). These groups mirror the barrier-remodeling and neuronal signaling axes identified across the full set of positively selected genes, indicating that the stage-biased subset is not functionally random. Nevertheless, because expression differences between adults and nymphs primarily reflect developmental regulation, this overlap identifies stage-biased candidates for functional follow-up and does not by itself demonstrate stage-specific adaptive function.

### 3.5. Stage-Biased Expression of Transcription Factor Families

**Figure 6:**
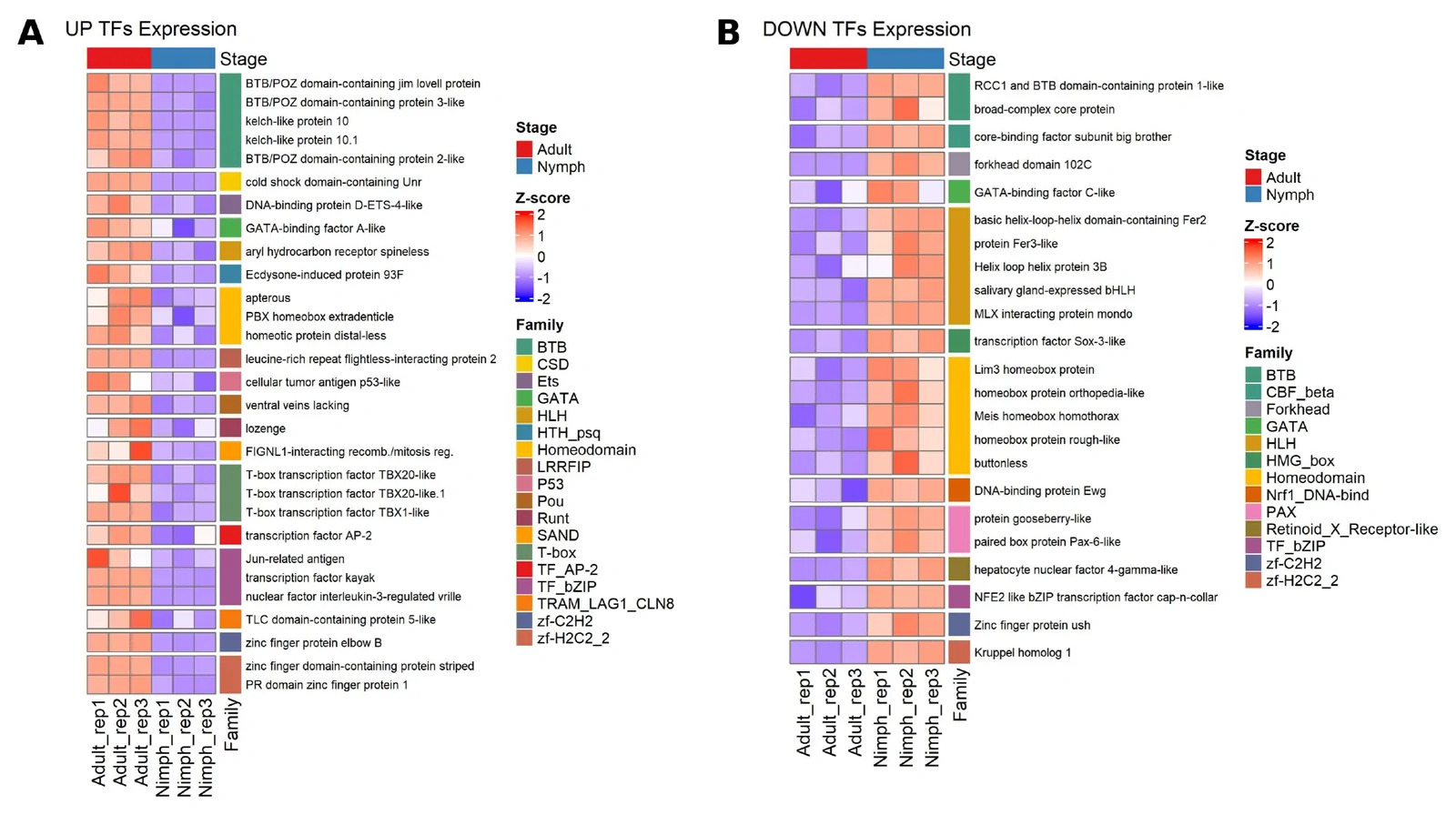
Stage-biased expression of differentially expressed transcription factors in *O. japonica*. (**A**) The 29 TF-encoding genes up-regulated and (**B**) the 23 down-regulated in adults relative to nymphs. Rows, genes (protein product names); columns, biological replicates (*n* = 3 per stage). Colors show row-scaled z-scores of normalized expression (red, high; blue, low).

Because the gene family analysis highlighted lineage-specific expansion of C2H2 zinc-finger and BTB/POZ transcription factor families, we examined the expression of annotated transcription factors between the nymph and adult stages (Figure 6). Numerous transcription factors were differentially expressed between the two stages, partitioning into an adult-biased set (Figure 6A) and a nymph-biased set (Figure 6B) and spanning a broad range of families, including C2H2 and H2C2 zinc-finger, BTB/POZ, homeodomain, basic helix-loop-helix (bHLH), bZIP, GATA, T-box, forkhead, and PAX factors. Adult-biased transcription factors included several BTB/POZ-domain and kelch-like proteins (jim lovell, BTB/POZ-domain proteins 2- and 3-like, kelch-like 10 and 10.1), consistent with the BTB/POZ expansion noted above, together with the ecdysone-responsive regulator Ecdysone-induced protein 93F, the xenobiotic-sensing bHLH factor Spineless (aryl hydrocarbon receptor), the homeodomain factors apterous, extradenticle, and Distal-less, T-box factors (*TBX1*- and *TBX20*-like), transcription factor *AP-2*, and the bZIP factors Jun-related antigen, kayak, and vrille. Nymph-biased transcription factors were dominated by developmental and metamorphosis-associated regulators, including the ecdysone-signaling factors broad-complex core protein and Krüppel homolog 1, multiple homeodomain factors (Lim3, orthopedia, homothorax, and rough), bHLH factors (Fer2, Fer3, and Mondo/MLX), the PAX factors gooseberry and *Pax-6*, the retinoid-X-receptor-like factor hepatocyte nuclear factor 4-γ, and the cap-n-collar bZIP factor *NFE2*, a conserved master regulator of xenobiotic detoxification. The predominance of ecdysone-responsive and homeobox regulators among the stage-biased transcription factors is consistent with the extensive developmental remodeling that accompanies the nymph-to-adult transition and links the lineage-specific expansion of zinc-finger and BTB/POZ families to their differential deployment across the life cycle.

## 4. Discussion

Biological invasions by phytophagous insects represent one of the most consequential threats to global agricultural productivity, native biodiversity, and ecosystem stability, with annual economic losses attributed to invasive arthropods exceeding tens of billions of dollars worldwide [72,73]. Central to this problem is the long-standing genetic paradox of invasion, whereby populations founded by few individuals and depleted of standing variation nevertheless adapt rapidly to unfamiliar hosts, climates, and chemical control regimes [74]. Resolving this paradox requires moving beyond basic genetic markers to full-genome analysis. Conventional population surveys simply miss the key drivers of adaptation, including gene duplication, coding sequence changes, and regulatory rewiring [75,76]. Comparative genomic frameworks now provide two complementary and widely applied instruments for this purpose. First, gene family expansion and contraction analyses quantify lineage-specific gains and losses of functional gene copies, thereby identifying the metabolic, sensory, and regulatory toolkits that distinguish successful invaders from their native-range relatives [43,44,77,78]. Second, branch site codon models, by contrast, detect episodic positive selection acting on individual loci along a focal lineage, revealing the fine-scale sculpting of protein function that accompanies ecological transitions [28,45]. Applied jointly, these approaches distinguish adaptation achieved through copy number from adaptation achieved through sequence change. Both approaches, however, detect evolutionary changes accumulated along the entire focal lineage; they characterize the long-term evolutionary background of the species and cannot, by themselves, distinguish ancient lineage-specific evolution from recent adaptation associated specifically with the invasion of Türkiye. Accordingly, the functional interpretations offered below should be read as hypotheses: candidate mechanisms that are consistent with the observed genomic patterns but that require functional validation and invasive-versus-native population comparisons for confirmation. Despite recent advances, the superfamily Fulgoroidea, particularly the family Ricaniidae, remains severely underrepresented in comparative genomic studies. This leaves critical gaps in our understanding of the molecular basis of invasion in this economically significant clade. The present study addresses this gap by applying an integrated comparative genomic framework to *O. japonica*, combining the first functional annotation of its genome with gene family dynamics modeling and genome-wide positive selection analyses to identify candidate molecular mechanisms potentially contributing to its invasive success.

CAFE analysis revealed 52 gene families with significant lineage-specific size changes in *O. japonica*, consisting of 24 expansions and 28 contractions. Notably, the functional composition between these two sets is markedly asymmetric. Expansions concentrated heavily on families mediating interactions with the external environment. Multiple major facilitator superfamily and sugar-transporter orthogroups were amplified. This pattern is consistent with the demands of an osmotically extreme, nitrogen-poor phloem diet, as well as with the dietary flexibility needed to switch between chemically distinct hosts like tea, hazelnut, and kiwifruit, although a direct functional link between these expansions and host use remains to be demonstrated. Furthermore, we observed an amplification of α/β-hydrolase-fold enzymes carrying ferulic acid esterase domains, glycoside hydrolase family 1 β-glucosidases, and carbon–nitrogen hydrolase/vanin-like enzymes. These expansions were supported by corresponding enrichments in hydrolase, galactosidase, and arylformamidase activities, alongside enriched tryptophan and kynurenine catabolism, consistent with an enhanced hydrolytic and redox capacity targeting plant-derived conjugates and their reactive intermediates; these represent candidate mechanisms that await functional validation.

Expansion of a pheromone-binding/OS-D chemosensory family is consistent with, though does not demonstrate, the broadened host recognition expected of a generalist, and comparative analysis in herbivorous Drosophilidae cautions that chemosensory repertoire size does not track dietary breadth in any simple way [79]. Two further expansions extend beyond feeding biology: serpins, the principal negative regulators of the extracellular protease cascades governing melanization and Toll signaling [80,81], provide a candidate mechanism for immune homeostasis under exposure to the novel entomopathogen communities of the invaded range; and a Methuselah-like GPCR family, members of which modulate longevity and thermal and oxidative stress tolerance across an ancient but arthropod-diversified receptor lineage [82]. Strikingly, the contracted set includes two families most frequently invoked to explain invasive success: cytochrome P450 and small heat-shock protein/α-crystallin, with monooxygenase and oxidoreductase activities correspondingly being enriched among reduced families. Neither contraction is as anomalous as it first appears. The CYP3 clan is the most evolutionarily unstable region of the arthropod CYPome, and detoxification-associated P450 clades are demonstrably more prone to both duplication and loss than physiologically constrained ones, indicating that repertoire size is a labile rather than a directional trait [83,84]. Likewise, HSP repertoires turn over rapidly among insect lineages, and thermal tolerance depends more on the inducibility and regulation of chaperone expression than on constitutive gene number [85,86]. We therefore interpret these contractions as evidence that copy number amplification of canonical phase I enzymes represents one route to xenobiotic tolerance rather than a universal signature of invasiveness, particularly since the regulatory machinery governing inducible detoxification—the cap-‘n’-collar factor NFE2/CncC and the xenobiotic-sensing receptor Spineless/AhR—remains intact and transcriptionally deployed [87,88]. Finally, the joint expansion of C2H2 zinc-finger and hAT/Hermes/THAP-domain families against contracting reverse transcriptase-containing orthogroups is consistent with ongoing genome repeat conflict, a dynamic implicated in generating adaptive variation in founder populations with depleted standing diversity [89]; this interpretation, too, remains a hypothesis to be tested.

Branch site analyses identified 122 candidate genes under significant lineage-specific positive selection along the *O. japonica* branch, of which 95 carried at least one high-confidence positively selected site. Strikingly, functional annotation revealed that these candidates are concentrated not in classical detoxification enzymes but in neuronal signaling (e.g., the planar cell polarity cadherin flamingo and the microtubule-associated protein futsch), epithelial barrier remodeling (*mucin-3A*, *mucin-17*, grainyhead, chitin synthase *chs-2*), and stress tolerance pathways. As an organizing framework, we group these candidates into three functional axes: host detection and neuronal excitability, xenobiotic/oxidative/proteostatic stress tolerance, and barrier remodeling. We stress that branch site tests detect selection accumulated along the species lineage; they do not establish that selection occurred during, or in response to, the recent invasion, host range expansion, or insecticide exposure, and the functional associations drawn below are therefore hypotheses. Nervous system functions emerge as the most consistently shared adaptive category across nine environmental gradients in invasive *Drosophila suzukii*, prompting the proposal of a broad adaptive role for neural evolution in insects [90], and comparable neuronal and signaling signatures accompany invasion in other polyphagous pests [91]; flamingo and futsch, canonical planar cell polarity and microtubule regulators of neural architecture [92], would fit this expectation in a species dependent on locating and accepting diverse hosts. The barrier-remodeling module is equally coherent: grainyhead is the conserved master regulator of cuticular and epidermal barrier formation, with functional conservation demonstrated in insects [93], while mucins are structural determinants of midgut peritrophic barrier integrity and permeability [94]. Both are plausible applied targets, since cuticular modification can confer penetration resistance by slowing insecticide entry [95]. Finally, selection on the chaperone Ric-3, which governs assembly and trafficking of nicotinic acetylcholine receptors and modulates neonicotinoid action in other systems [96,97], generates a testable pharmacological hypothesis rather than a demonstrated mechanism. Collectively, these signatures are consistent with the view that adaptive evolution in this lineage has proceeded through multiple, non-interchangeable molecular routes [98].

The intersection of the selection results with the developmental transcriptome should likewise be interpreted with caution. Positive selection detected in coding sequence may act on protein properties—localization, binding affinity, stability, or turnover—without altering mRNA expression, and the nymph-to-adult transition itself drives profound, genome-wide transcriptional change; that only 15 of the 122 candidates were differentially expressed between stages is therefore unsurprising and carries no adaptive implication on its own. We regard this intersection primarily as a prioritization tool. Notably, the stage-biased candidates cluster qualitatively into epithelial-barrier components (the mucins, chitin synthase *chs-2*, and *mesh*), membrane-associated neuronal and signaling proteins (Ric-3, the ryanodine receptor, and neuroligin-4), and transcriptional or chromatin regulators (pygopus, AT-rich interactive domain-containing protein 1A, and box A-binding factor), echoing the barrier and neuronal axes recovered across the full candidate set and suggesting concrete, stage-aware entry points for functional work. Independently of the selection analysis, the transcription factor complement recovered the canonical hemimetabolan metamorphic switch, providing internal validation of the dataset; the nymph-biased set was dominated by broad-complex and Krüppel homolog 1, the juvenile hormone-dependent effectors that repress adult morphogenesis, whereas the ecdysone-induced factor E93 was adult-biased, matching precisely the MEKRE93 configuration expected as juvenile hormone declines at the final molt [99].

Two superimposed patterns bear on invasion biology. First, adult-biased factors included BTB/POZ-domain and kelch-like proteins from families identified as expanded by CAFE, indicating that gene amplification is accompanied by differential ontogenetic deployment. This pattern is consistent with regulatory co-option, although adult–nymph expression differences alone cannot demonstrate co-option or adaptive function, and this interpretation remains to be tested. Second, xenobiotic sensing exhibits clear stage partitioning: NFE2/CncC was nymph-biased, whereas Spineless/aryl hydrocarbon receptor was adult-biased. This pattern aligns with the inducible detoxification hypothesis advanced above and with functional evidence that insect AhR regulates detoxification gene expression and insecticide susceptibility [100]. Barrier components follow the same logic; mesh is nymph-biased [101] while chs-2 is adult-biased, implying stage-dependent differences in barrier composition. This structural shift has potential management relevance. Chitin pathway genes are validated RNAi targets in other planthoppers, where nanoparticle-formulated dsRNA sprays achieve field-relevant mortality [102,103,104], suggesting that delivery efficacy would depend on which developmental stage is targeted; we note, however, that these management implications are extrapolated from other species and have not yet been tested in *O. japonica*. These inferences also remain bounded by whole-body sampling, in which stage contrasts partly reflect tissue composition.

## 5. Conclusions

We report the first functional annotation of the *O. japonica* genome and the first comparative genomic characterization of this invasive planthopper. Lineage-specific expansions in transport, hydrolytic, chemosensory, and stress-signaling families, set against contraction of cytochrome P450 and small heat-shock proteins, indicate that metabolic gene content has been reshaped selectively rather than uniformly amplified. Branch site tests identified 122 candidate genes under lineage-specific positive selection, whose annotations converge on host detection, stress tolerance, and barrier remodeling, several of which are also stage-biased in expression. Because these signatures reflect evolution along the species lineage, they cannot yet be attributed to the recent invasion itself; rather, they delineate candidate genes and gene families potentially associated with invasion-related traits, whose roles now require functional validation and comparisons between native and invasive populations. Together, these resources and candidates provide a genomic foundation for evolutionary studies and for the development of targeted management strategies against this emerging pest.

## Figures and Tables

**Table 1:** The assembly and annotation statistics of the *O. japonica* genome.

Metric	Value
Genome Size (bp)	1,500,773,387
Genome GC (%)	31.93%
Total Sequences in FASTA	197
Chromosomes Mapped	12
Organelle/Mitochondrion	1
Unplaced Scaffolds/Contigs	184
Total Genes	15,243
Protein-Coding Genes	12,402
LncRNA	784
ncRNA	1
rRNA	236
snoRNA	45
snRNA	57
tRNA	1718
Total mRNAs	18,008
Total Exons	166,644
Average Exon Length (bp)	244.35
Total CDS Features	149,450
Total Proteins Predicted	17,652
Average Protein Length (aa)	546.50

## Data Availability

All raw genomic and RNA-Seq sequencing data generated during this study have been deposited in the NCBI Sequence Read Archive (SRA) database under BioProject accession number PRJNA1504096.

## Supplementary Materials

The following supporting information can be downloaded at: https://www.mdpi.com/article/10.3390/insects17090932/s1, Table S1: The sequencing results obtained from Oxford Nanopore long-read sequencing of *O. japonica* HMW DNA. Table S2: The result of the sequencing short-read obtained from the DNBSEQ-G400 sequencing platform of *O. japonica* HMW DNA. Table S3: RNA-seq alignment statistics. Table S4: BOLD Species Identification engine search result for the COI barcode region of *O. japonica*. Table S5: Quality metrics of the clean RNA-seq reads after quality control. Table S6: OMArk proteome assessment of the *O. japonica* annotation against the Paraneoptera reference lineage. Table S7: Significantly expanded and contracted gene families in *O. japonica* identified by CAFE analysis (*p* < 0.05). Each row corresponds to an orthogroup, annotated with its associated protein domain(s) (Pfam and InterPro identifiers and descriptions). The numeric columns give the inferred change in gene copy number along the terminal branch of *O. japonica* and of the 20 comparator species, where positive values indicate expansion and negative values indicate contraction relative to the reconstructed ancestral state. Table S8: Genes under positive selection in *O. japonica* identified by branch site model analysis (codeml, PAML), with *O. japonica* as the foreground branch. For each gene, the table reports the likelihood ratio test *p*-value, the Benjamini–Hochberg-adjusted *p*-value (FDR < 0.05), the estimated ω (dN/dS) for the positively selected site class, and the Bayes Empirical Bayes (BEB) posterior probabilities of individual positively selected sites. Genes are organized into those carrying high-confidence positively selected sites (BEB > 0.95) and those significant at the branch level without individually significant sites, with a per-site breakdown provided in a separate sheet. Gene symbols and product names follow the corresponding NCBI RefSeq orthologs.

## Abbreviations

The following abbreviations are used in this manuscript:

BOLD	Barcode of Life Data system
bp	Base pair
°C	Celcius
DEG	Differentially expressed gene
DNA	Deoxyribonucleic acid
*g*	Relative centrifugal force
Gb	Gigabase
GC	Guanine–cytosine content
HMW	High molecular weight
kb	Kilobase
min	Minute
ml	Milliliter
NCBI	National Center for Biotechnology Information
padj	Adjusted *p*-value
RIN	RNA integrity number
RNA	Ribonucleic acid
µl	Microliter
