# Supplementary material for: A Comparative Genomic Assessment of Invasive Potential in the Planthopper Orosanga japonica (Hemiptera: Ricaniidae)
Source: Insects. 2026 Sep 5;17(9):932. doi: 10.3390/insects17090932 (PMC13607038; doi:10.3390/insects17090932)
Supplement: Supplementary file 1 [file insects-17-00932-s001.zip › Supplementary_Files_new.pdf]

Supplementary Information for

A Comparative Genomic Assessment of Invasive Potential in the Planthopper Orosanga japonica (Hemiptera: Ricaniidae)

Yusuf Ulaş Çınar [1], Mehmet Ali Balcı [1], Onur Obut [1], Tuana Öğretici [1], Selahattin Barış Çay [1], Hüsna Yağmur Dural [1], Fatih Dikmen [2], Yakup Bakır [3,*] and Vahap Eldem [2,*]

[1] Institute of Graduate Studies in Sciences, Istanbul University, Istanbul, 34134, Türkiye

[2] Department of Biology, Faculty of Science, Istanbul University, Istanbul 34134, Türkiye

[3] Department of Medical Laboratory Techniques, Vocational School of Health Services, Atlas University, Istanbul 34403, Türkiye

[*] **Correspondence:** yakup.bakir@atlas.edu.tr (Y.B.); vahap.eldem@istanbul.edu.tr (V.E.)

Webpage: https://avesis.istanbul.edu.tr/vahap.eldem

Office Phone: +90 212 455 5700 (Extension: 15086)

Supplementary Tables

Table S1. The sequencing results obtained from Oxford Nanopore long-read sequencing of *O. japonica* HMW DNA.

Metric	Value
**Mean read length (bp)**	9063
**Mean read quality**	13.3
**Median read length (bp)**	2534
**Median read quality**	14.1
**Read length (bp)**	13599
**Number of reads**	727605
**Read length N50 (bp)**	31857
**Total bases (bp)**	6594374110

Table S2. The result of the sequencing short-read obtained from DNBSEQ- G400 sequencing platform of *O. japonica* HMW DNA.

File Name	Number of Reads	Total Nucleotides	Read Length (bp)	Q20 (%)	Q30 (%)	GC (%)
**HS1_1.fq.gz**	318047568	47707135200	150	97.41	90.70	32.84
**HS1_2.fq.gz**	318047568	47707135200	150	95.67	87.55	33.04
Total	636095136	95414270400				

Table S3. RNA-seq alignment statistics.

Sample	Stage	Primary Mapped Reads	Mapping Rate (%)
**Adult_rep1**	Adult	26463563	91.30
**Adult_rep2**	Adult	26465803	91.32
**Adult_rep3**	Adult	26472561	91.31
**Nimph_rep1**	Nimph	22141652	91.06
**Nimph_rep2**	Nimph	22153487	91.07
**Nimph_rep3**	Nimph	22145159	91.07

Table S4. BOLD Species Identification engine search result for the COI barcode region of *O. japonica*.

PID [BIN]	Phylum	Class	Order	Family	Genus	Species	ID%
**GMGKA2489-21**	Arthropoda	Insecta	Hemiptera	Ricaniidae	*Orosanga*	*O. japonica*	100
**GMGKB1390-20**	Arthropoda	Insecta	Hemiptera	Ricaniidae	*Orosanga*	*O. japonica*	100
**GBAAZ28456-24**	Arthropoda	Insecta	Hemiptera	Ricaniidae	*Orosanga*	*O. japonica*	100
**GBMNE23131-21**	Arthropoda	Insecta	Hemiptera	Ricaniidae	*Orosanga*	*O. japonica*	99.85
**GBAAZ28457-24**	Arthropoda	Insecta	Hemiptera	Ricaniidae	*Orosanga*	*O. japonica*	99.84
**GBAAZ28458-24**	Arthropoda	Insecta	Hemiptera	Ricaniidae	*Orosanga*	*O. japonica*	99.84
**LPGES15139-25**	Arthropoda	Insecta	Hemiptera	Ricaniidae	*Orosanga*	*O. japonica*	99.70
**GMGKA2515-21**	Arthropoda	Insecta	Hemiptera	Ricaniidae	*Orosanga*	*O. japonica*	99.69
**GMGKA2509-21**	Arthropoda	Insecta	Hemiptera	Ricaniidae	*Ricania*	*R. simulans*	95.53

**GBAAZ29646-24**	Arthropoda	Insecta	Hemiptera	Ricaniidae	*Ricania*	*R. simulans*	94.23

Table S5. Quality metrics of the clean RNA-seq reads after quality control.

Sample	Number of Sequences	Total Length (bp)	Lentgh (base)	Q20(%)	Q30(%)
**Adult_rep1_R1**	13913324	1921919449	150	95,37	88,66
**Adult_rep1_R2**	13913324	1917588549	150	96,71	91,32
**Adult_rep2_R1**	15217699	2102112903	150	95,37	88,66
**Adult_rep2_R2**	15217699	2097425697	150	96,71	91,31
**Adult_rep3_R1**	14348116	1982079628	150	95,37	88,65
**Adult_rep3_R2**	14348116	1977599077	150	96,71	91,31
**Nimph_rep1_R1**	12402581	1684284796	150	95,42	88,73
**Nimph_rep1_R2**	12402581	1679449038	150	97,08	91,93
**Nimph_rep2_R1**	13132144	1783411732	150	95,42	88,73
**Nimph_rep2_R2**	13132144	1778346248	150	97,08	91,92
**Nimph_rep3_R1**	10943454	1486070289	150	95,42	88,74
**Nimph_rep3_R2**	10943454	1481852127	150	97,09	91,93
Total	159914636	21892139533			

Table S6. OMArk proteome assessment of the *O. japonica* annotation against the Paraneoptera reference lineage.

Category	Count	Percentage
**COMPLETENESS (conserved HOGs, n = 4,585)**		
**Single-copy**	4,239	92.45%
**Duplicated**	98	2.14%
**Missing**	248	5.41%
**CONSISTENCY (predicted proteome, n = 12,402)**		
**Consistent**	8,866	71.49%
**Inconsistent**	1,603	12.93%
**Unknown**	1,781	14.36%
**Potential contaminants**	152	1.23%
**SPECIES COMPOSITION**		
**Cimicomorpha (main clade)**	7,624	61.47%
**Pezizomycotina (contaminant 1)**	161	1.30%
